# Right ventricular strain, torsion and synchrony in healthy subjects using 3D spiral cine DENSE

**DOI:** 10.1186/1532-429X-18-S1-O83

**Published:** 2016-01-27

**Authors:** Jonathan D Suever, Gregory J Wehner, Christopher M Haggerty, Linyuan Jing, David Powell, Sean M Hamlet, Jonathan D Grabau, Dimitri Mojsejenko, Kristin N Andres, Moriel Vandsburger, Brandon K Fornwalt

**Affiliations:** 1 grid.280776.c0000000403941447Institute for Advanced Application, Geisinger Health System, Danville, PA USA; 2 grid.266539.d0000000419368438Pediatrics, University of Kentucky, Lexington, KY USA; 3 grid.266539.d0000000419368438Biomedical Engineering, University of Kentucky, Lexington, KY USA; 4 grid.266539.d0000000419368438Electrical and Computer Engineering, University of Kentucky, Lexington, KY USA; 5 grid.266539.d0000000419368438College of Medicine, University of Kentucky, Lexington, KY USA; 6 grid.266539.d0000000419368438Physiology, University of Kentucky, Lexington, KY USA

## Background

Mechanics of the left ventricle (LV) are important indicators of cardiac function. The role of right ventricular (RV) mechanics is unknown due to the technical limitations of imaging its thin wall and complex geometry and motion. By combining 3D Displacement Encoding with Stimulated Echoes (DENSE) with a local coordinate system analysis, it is possible to quantify RV strain, torsion, and synchrony. In this study we sought to characterize RV mechanics in healthy individuals and compare these values to their LV counterparts.

## Methods

**Figure 1:**
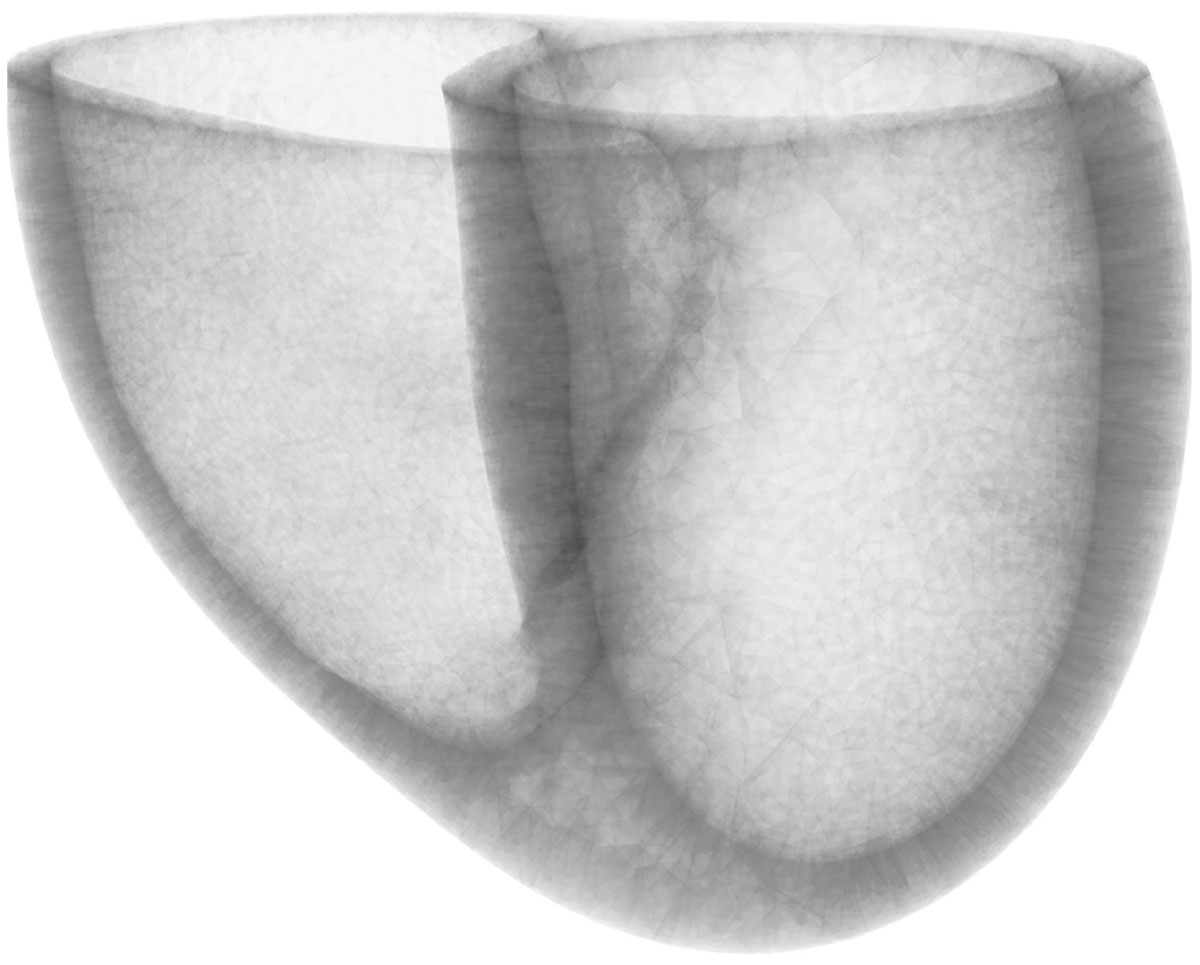
**Dual Ventricular Tetrahedral Mesh**. A tetrahedral mesh of the myocardium was computed using the epicardial contours as well as the endocardial contours drawn on both the left and right ventricles. This mesh was used to define a local coordinate system for computing the strain tensor.

Spiral cine DENSE was performed on 40 healthy subjects (age: 27 ± 8; 53% female) at 3T (Siemens Trio). Short-axis images spanning both ventricles at end-diastole were acquired with displacements encoded in three dimensions. Acquisition parameters included: 12 spiral interleaves, 360 × 360 mm2 FOV, 180 × 180 image matrix, 8 mm slice thickness, TE/TR = 1.08/17 ms, 0.04 cycles/mm encoding frequency. For each cardiac frame, the 3D displacements were fit to continuously differentiable radial basis functions, allowing for computation of the 3D Cartesian Lagrangian strain tensor at any myocardial point. The geometry of the RV was extracted via a surface fit to manually drawn endocardial contours (Figure 1). Throughout the RV, the local longitudinal direction was tangent to the surface and pointed toward the manually defined RV apex. The local circumferential direction was tangent to the surface and orthogonal to the local longitudinal direction. The Cartesian strain tensors were then transformed to the local coordinate system. Regional peak RV circumferential strain (Ecc), longitudinal strain (Ell), and torsion were reported. Comparable analysis was performed for the LV. Regional activation times (expressed as percent cardiac cycle (%CC)) were determined by performing cross-correlation between regional 2nd principal strain curves and an average curve obtained from both ventricles. Synchrony was assessed as the standard deviation of all regional activation times within each ventricle.

## Results

**Figure 2:**
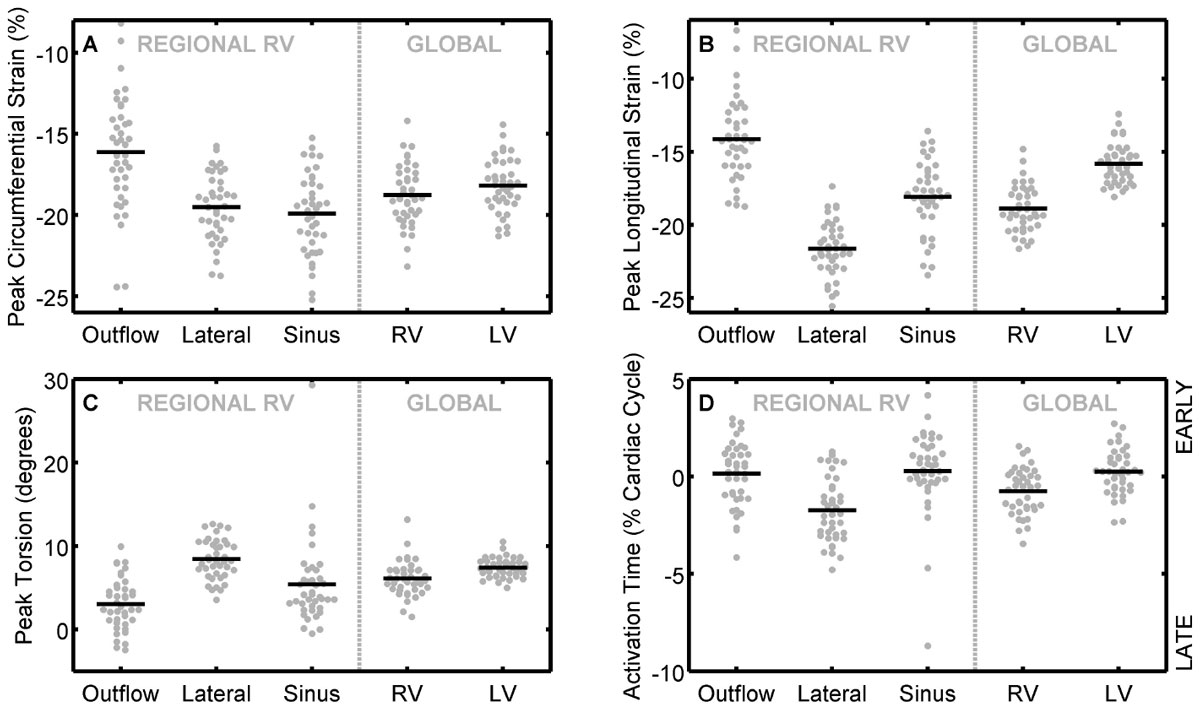
**Regional Strain, Torsion, and Synchrony in the RV**. The outflow region of the RV demonstrated lower Ecc compared to all remaining segments and global Ecc was comparable between the LV and RV (A). Ell varied considerably around the circumference of the RV (14 - 22%) with global Ell being higher in the RV relative to the LV (B). RV torsion values were similar to those observed in the LV (C) and activation times varied throughout the RV with the RV contracting after the LV and the lateral wall of the RV contracting last (D).

Ecc varied regionally within the RV with the lowest values (16%) in the outflow region (Figure 2A). Ell varied considerably around the circumference of the RV (14 - 22%) with global Ell being higher in the RV relative to the LV (Figure 2B). RV torsion was found predominantly in the lateral region and was comparable to LV torsion (Figure 2C). Regional activation times indicated that the RV as a whole contracted later than the LV with the lateral wall of the RV contracting last (Figure 2D). Interestingly, the RV was found to be slightly more synchronous than the LV (2.7 ± 1.0 vs. 3.4 ± 1.1 %CC).

## Conclusions

3D spiral cine DENSE is capable of resolving both the complex geometry and 3D motion of the RV. In healthy subjects, regional variations in Ecc and Ell exist within the RV, however they have comparable global magnitudes. RV torsion was similar to torsion seen in the LV and the RV was found to contract later but more synchronously than the LV.

